# Characterizing the most demanding passages of kinematic and mechanical activity in elite football: a multifactorial approach

**DOI:** 10.5114/biolsport.2024.134756

**Published:** 2024-03-17

**Authors:** Farzad Yousefian, Abdullah Zafar, Dan Fransson, Magni Mohr, João Brito, Bruno Travassos

**Affiliations:** 1 Research Centre in Sports Sciences, Health Sciences and Human Development (CIDESD), Department of Sport Sciences, University of Beira Interior, Covilhã, Portugal; 2 Portugal Football School, Portuguese Football Federation, Oeiras, Portugal; 3 Department of Kinesiology, University of Waterloo, Waterloo, Canada; 4 Center for Health and Performance, Department of Food and Nutrition and Sports Science, University of Gothenburg, Gothenburg, Sweden; 5 Department of Sports Sciences and Clinical Biomechanics, SDU Sport and Health Science Cluster (SHSC), University of Southern Denmark, Odense, Denmark; 6 Center of Health Science, Faculty of Health, University of the Faroe Islands, Tórshavn, Faroe Islands

**Keywords:** Most intense periods, GPS, Match demands, Performance, Team sports

## Abstract

This study investigated the 5-minute most demanding passages (MDP) of kinematic (distance covered) and mechanical (acceleration and deceleration) activities in elite male football according to multifactorial criterion performance variables. Global positioning systems data were collected from 39 players across 45 matches in the Swedish first division (n = 329 observations). The multifactorial kinematic variable (MDPk) was composed of the concurrent distances covered at moderate-speed running, high-speed running, and sprinting distances, and the multifactorial mechanical (MDPm) considered the concurrent occurrences of high-intensity acceleration and deceleration activities. A moving average method was used to identify the MDP across a 5-minute period. The frequency distribution of the multifactorial variables, and differences in the time of occurrence and magnitude between multifactorial performance variables and their univariate constituent variables were investigated. Frequency distribution analysis revealed MDPk and MDPm peaked in the first 5 min of the match (MDPk: χ**^2^** (15, N = 329) = 135.88, p ≤ 0.001, *W*: 0.64, 115.99 ± 32.7 s; MDPm: χ**^2^** (15, N = 329) = 31.02, p ≤ 0.001, *W*: 0.31, 101.21 ± 25.1 s; p ≤ 0.0004). Within each half, differences in the MDP commencement time between the multifactorial variables and their respective discrete univariate constituent variables were trivial to small (MDPk effect size (ES): 0.04–0.21; MDPm ES: 0.02–0.11). Linear mixed model analysis demonstrated that the MDP magnitude of multifactorial variables were approximately 8–28% and 8–21% lower across the match and halves, respectively, compared to the MDP of their respective univariate constituent variables (p ≤ 0.001; *r* = 0.26–0.62). The greatest differences between the respective multifactorial and discrete constituent variables were observed for sprinting (-28%) and high-intensity acceleration (-22%). The results reveal that the MDP is distinguished based on peak kinematic and mechanical demands, which occur at discrete periods and exhibit distinct locomotor profiles across the match and within each half. Practitioners should consider the methods of identifying the MDP, as the selection of univariate and multifactorial kinematic and mechanical performance variables can impact MDP characterization, which can qualify the designing of bespoke training protocols.

## INTRODUCTION

In recent years, numerous studies have employed tracking system microtechnologies, such as global positioning systems (GPS), to describe the physical demands of elite football players during matches [1], offering insights for training design [2]. Given the stochastic nature of football, physical demands on players continuously fluctuate in magnitude, effort, and time due to changing game dynamics [3, 4], leading to differential intensity patterns and the most demanding passages (MDP) of physical activity [5–8]. Thus, a comprehensive understanding of the MDP can inform effective training design to ensure optimized performance and minimized risk to injury when preparing players for competition [6, 9].

Current research supports the use of a continuous moving average method for robust MDP identification during matches compared to fixed length method [5, 8, 9, 10]. A fixed length method splits the match into fixed periods (e.g., for 5 min periods: 0–5 min, 5–10 min, etc.), while a moving average method considers the maximal load across a given time point second by second (e.g., a 5 min period at 1Hz sampling frequency would include 300 data points: 0–299, 1–300, 2–301, etc.). For instance, Varley et al. [8] found a 25% underestimation of peak high-intensity running (HIR; > 15 km/h) distance using fixed length windows compared to a moving average approach. Similarly, more recent studies have revealed significant underestimations of the MDP of total distance and high-speed running distance by as much as 7% and 22%, respectively by fixed length compared to a moving average method [5, 7].

Several researchers have explored MDP of external load performance variables (running distances and accelerations/decelerations) across various thresholds (e.g., > 15 km/h, > 19.8 km/h, > 25.2 km/h, > 3 m/s**^2^**, < -3 m/s**^2^**) and time windows (1 to 10 min) [5, 7, 8, 9, 11]. For example, the 5 min MDP in elite male football have been reported for total distance (~130–150 m/min), moderate-speed running (> 15 km/h; 35.4 ± 18.2 m/min), high-speed running (> 19.8 km/h; ~20 m/min), average acceleration/deceleration (~0.60 m/s**^2^**), and the number of high-intensity acceleration (> 3 m/s**^2^**; 2 n/min) and deceleration (< -3 m/s**^2^**; 2 n/min) [5, 7, 8, 11, 12, 13]. The temporal distribution of MDP has also been investigated recently, with the authors of the studies reporting that highest frequencies of peak total distance and acceleration load (average acceleration/deceleration) occur within the first 15 min of the match and each half, across peak 1 min to 10 min durations, whereas HSR distribution is uniformly distributed [12, 14, 15]. When analyzed according to 3 min durations, Novak et al. [16] reported peak total distance and sprinting distance were associated with earlier and later occurrences within the half, respectively, while no association with match half was observed for HSR [16]. However, methodological differences, including the use of different performance variables and moving average time windows limit direct comparisons between studies.

More specifically, existing research examines MDP individually as discrete univariate metrics, limiting their application in training session design and neglecting MDP periods whereby multiple variables interact and may occur concurrently [9, 16, 17]. Translating findings to training is thus restricted, particularly to ensure the intended transfer to the context of performance. Thus, it may be relevant to identify the MDP by using a multifactorial criterion variable which encompasses the concurrent occurrence of different high-intensity velocity-based (kinematic) or mechanical (rate of change in velocity; acceleration and deceleration) external load constituent variables simultaneously. This approach may be supported by a theoretical framework proposed by Vanrenterghem et al., [18], whereby kinematic (measures of distance across speed thresholds) and mechanical (measures of acceleration/deceleration magnitudes) loads are uncoupled to better understand their relative contributions underpinning different load-adaptation pathways. In effect, targeting the specific pathways, in terms of training prescription, and monitoring their relative contributions to the locomotor profile of players during the match, could better inform player performance monitoring and preparation. The relationship between the multifactorial most demanding kinematic (MDPk) and mechanical (MDPm) variables and the MDP of their respective constituent variables as discrete variables can be investigated in terms of their timing, interaction, and magnitude. In fact, studies highlight the effectiveness of sport-specific conditioning drills (e.g., small-sided games) for high transfer of demands between training sessions and performance context according to coach-intended game dynamics compared to specific/isolated running drills (e.g., interval and repeated sprint training) [9, 17, 19, 20]. Therefore, a multifactorial analysis of MDP may be more relevant in performance settings through enhancing performance monitoring strategies of players during such commonly applied training drills and provide further insight towards improving the methodologies and application of the MDP in elite football.

Thus, a multifactorial analysis approach based on capturing concurrent MDP of discrete high-intensity kinematic (MDPk) and mechanical (MDPm) variables within match dynamics is suggested. This study proposes and tests a multifactorial analysis to identify and characterize 5-minute MDP of high-intensity activity in elite male football based on concurrent kinematic and mechanical performance variables. Based on this approach, the aims of the study were to describe the frequency distribution of the respective multifactorial performance variables MDPk and MDPm; and to understand the relationship between the MDP of the multifactorial metrics and their respective discrete univariate variables in terms of their timing of occurrence and relative magnitude. We hypothesize a similar temporal distribution for MDPk and MDPm, with a greater frequency of MDP cases occurring at the start of the match and within each half. Furthermore, we hypothesize that there will be small differences in terms of timing and magnitude between univariate and multivariate constituent variables, with the MDP instances occurring within relatively close proximity.

## MATERIALS AND METHODS

### Study Design

A retrospective study was conducted using tracking data collected from two teams in the Swedish first division (*Allsvenskan*) during the 2020 and 2021 seasons. Prior to the start of the investigation, players were verbally informed of the aims of the study and permission to use collected data was obtained from the participating players and clubs. Ethical approval of the study was obtained from the ethics review committee of the University of Gothenburg (#2021-05974-01), and the study was conducted in accordance with the principles of the Declaration of Helsinki.

### Participants

A total of 39 professional male football players (mean ± standard deviation: age: 27.9 ± 4.5 years; height: 182. 6 ± 5.9 cm; weight: 74.7 ± 6.0 kg) participated in the study. Data were collected from 45 competitive matches of the 2020 (7 matches) and 2021 (38 matches) seasons, resulting in 329 match observations (mean ± standard deviation: 8.4 ± 6.8 observations per player; range: 1–24). Only outfield players who completed the total duration of the match were included in the study.

### Procedure

Match-play locomotor data was collected using 10-Hz GPS devices (Catapult Vector S7, Catapult Sports, Melbourne, Australia). The GPS devices have been reported as valid and reliable to measure force-velocity profiles [21] and the inter-device reliability for distance, peak velocity and average acceleration was reported as *good* (%CV: 0.1–3.9%) [22]. The 10 Hz devices demonstrated to be reliable and acceptable for tracking soccer specific movement including instantaneous velocity, maximum instantaneous velocity, as well acceleration and decelerations [23, 24]. Prior to the start of every match, all devices were calibrated according to the manufacturer’s instructions, and placed between the players’ shoulder blades in tightly fitted vests during the match. Throughout the study, each player wore the same GPS unit to avoid inter-unit variation. Following each match, raw GPS data were downloaded using the manufacturer’s proprietary software and analyzed using customized Microsoft Excel spreadsheets and Python 3.9 software. Data underwent satellite connectivity check before raw data analysis; instances with fewer than eight connected satellites were excluded.

### Performance Variables

A moving average method was used to identify the MDP across 5 min considering univariate kinematic (running distance covered) and mechanical (acceleration/decelerations) performance variables including moderate-speed running (MSR; 15–19.8 km × h**^−1^**), high-speed running (HSR; > 19.8–25.2 km × h**^−1^**), and sprinting (SPR; > 25.2 km × h**^−1^**) distances, as well as high-intensity acceleration (ACC; ≥ 3 m × s**^−2^**) and high-intensity deceleration (DEC; ≤ -3 m × s**^−2^**) metrics, in accordance with previous studies [11, 25]. The MDPk metric was identified based on the total distance covered at running speed > 15 km h**^−^**1, thus consisting of the maximal sum of the moving average across a 5 min period of MSR, HSR, and SPR. Alternatively, the MDPm metric was identified based on the maximal absolute sum of the moving average across a 5 min period of high-intensity acceleration and deceleration efforts (≥ 3 m × s**^−2^**). The selected 5 min duration, as well as the kinematic velocity thresholds and mechanical acceleration thresholds were selected in accordance with previous related studies [11, 15]. Any additional time at the end of each half was removed prior to the analysis. The moving average included a 300 s window, based on the 10 Hz sampling frequency of the GPS devices, therefore, any period at the end of each half with less than 300 s was not included in the analysis [12]. The frequency distribution of MDPk and MDPm were analyzed based on their respective peak period commencement time and binned into discrete 5 min periods for each half and the match. Differences in the commencement time between MDPk and MDPm and their respective univariate constituent variables were investigated within halves and the match. Subsequently, magnitude differences between MDPk and MDPm and their respective constituent variables were analyzed across halves and the match.

### Statistical Analyses

The frequency distribution of MDPk and MDPm across the match and for each half were assessed using Pearson chi-square Goodness-of-Fit tests. The magnitude of statistical difference from a hypothetical equal distribution was assessed using Cohen’s *W* effect size. Binomial tests conducted pairwise comparisons of 5 min intervals following chi-square analysis. Bonferroni adjustments were applied to control for multiple comparisons and type I error, yielding adjusted α-levels of p ≤ 0.0004 for across match and p ≤ 0.002 for within-half analyses. Magnitude of significance was evaluated using Cohen’s *h* effect size [26].

Subsequently, the MDP of univariate metrics were compared with the MDPk and MDPm regarding their occurrence in the period within each half and across the match using standardized mean differences and ± 95% confidence intervals (± 95%CI). The magnitude of the differences was interpreted according to Cohen’s *d* effect size criteria: 0.2–0.5, small; 0.5–0.8, medium; and > 0.8, large.

Finally, differences in magnitude between the MDPk and MDPm and their respective constituent variables for each half and the match were assessed using linear mixed models. For each model, normality of the residuals was evaluated using a combination of the Kolgomorov-Smirnoff test and inspection of the Q-Q plots. In all models, the ‘player’ and ‘match’ were specified as random effects and fixed effects included match half, and ‘period’ (i.e., the peak performance variable as either a univariate or multifactorial constituent variable) [16]. Non-normally distributed data were log-transformed to reduce the non-uniformity of error and back-transformed in the presented results. Where significance was detected, multiple pairwise comparisons were assessed using Bonferroni *post-hoc* tests. Effect size correlations (*r*) were calculated from the linear mixed model *t* statistics and interpreted based on the following criteria: < 0.1, trivial; 0.1–0.3, small; 0.3–0.5, moderate; 0.5–0.7, large; 0.7–0.9, very large [27]. The % coefficient of variation (%CV) was also calculated as the standard deviation divided by the mean multiplied by 100%. Data are presented as mean ± SD, and the α-level was set at *P*≤0.05, unless otherwise stated. All statistical analyses were performed using IBM SPSS for Windows statistics version 25.0 (IBM Corp., Armonk, NY, United States).

## RESULTS

### Frequency distribution

**FIG. 1:**
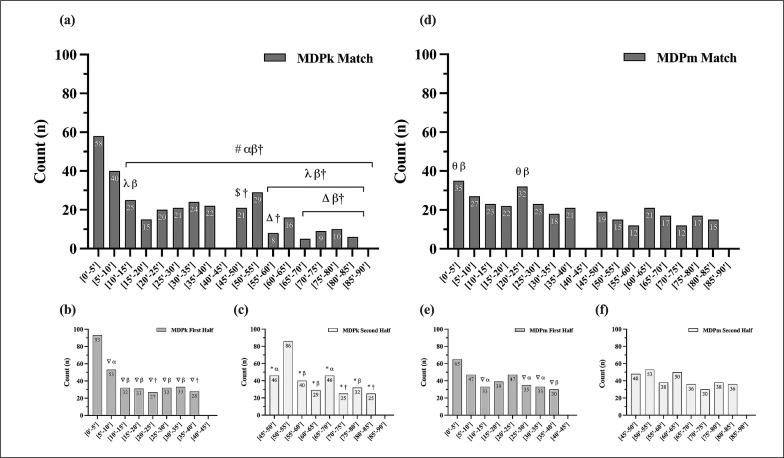
Frequency distribution of MDPk across the (a) match, (b) first half, and (c) second half, and MDPm across the (d) match, (e) first half, and (f) second half. # Significantly different to [0’-5’] (p ≤ 0.0004), λ Significantly different to [5’-10] (p ≤ 0.0004), $ Significantly different to [65’-70’] (p ≤ 0.0004); Δ Significantly different to [50’-55’] (p ≤ 0.0004); θ Significantly different to [55’-60’] and [70’-75’] (p ≤ 0.0004), ∇ Significantly different to [0’–5’] (p ≤ 0.002). * Significantly different to [50’-55’] (p ≤ 0.002). Effect size (*h*): 0.2–0.5, small (α); 0.5–0.8, medium (β), > 0.8, large (†).

The frequency distributions of MDPk and MDPm across the match and for each half are presented in Figure 1. MDPk showed large significant differences across the match (χ**^2^** (15, *N* = 329) = 135.88, p ≤ 0.001, *W*: 0.64) and in the first half (χ**^2^** (7, *N* = 329) = 86.05, p ≤ 0.001, *W*: 0.51). Medium effect size differences were observed in the second half (χ**^2^** (7, *N* = 329) = 68.40, p ≤ 0.001, *W*: 0.46). MDPm showed medium effect size differences across the match (χ**^2^** (15, *N* = 329) = 31.02, p ≤ 0.001, *W*: 0.31) and small differences only in the first half (first half: χ**^2^** (7, *N* = 329) = 22.78, p ≤ 0.001, W: 0.26; second half: χ**^2^** (7, *N* = 329) = 11.26, p = 0.26).

The highest frequency of MDPk and MDPm was observed in the first 5 min of the match (MDPk: ~18% of total cases; 101.21 ± 25.1 s; MIPm: ~11%; 115.99 ± 32.7 s. Within the halves, the highest MDPk frequency was observed in the first 5 min of each half (first half: ~28%; 111.47 ± 19.8 s; second half: ~26%; 3159.17 ± 19 s), with ~54% and ~52% of total cases occurring in the first 15 min of each half. Regarding the MDPm, the highest frequency occurred in the first 5 min of the first half (~20%; 105.18 ± 23.3s), and in the second 5 min period of the second half (~16%; 3152.14 ± 22.4s), with ~44% and ~42% of total cases occurring in the first 15 min of each respective half.

### Timing of occurrence

**FIG. 2:**
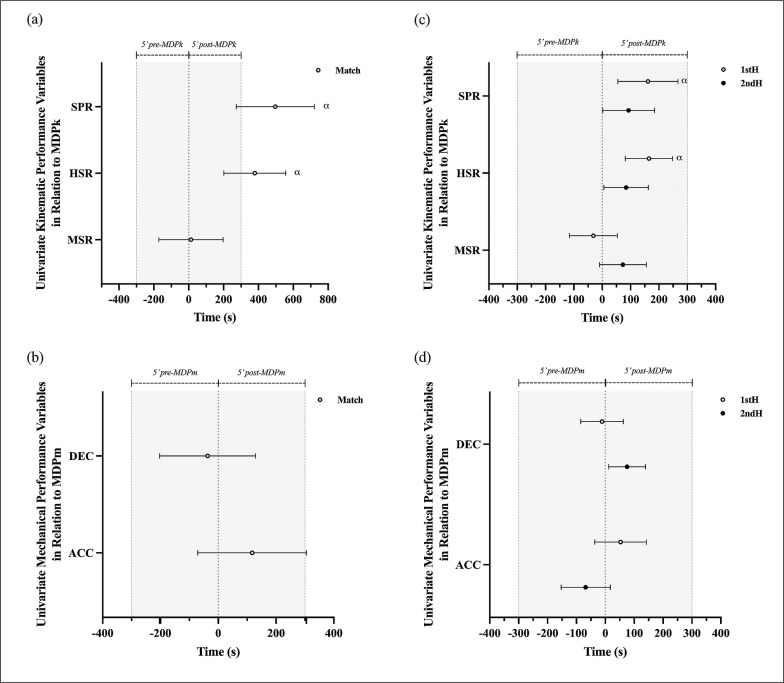
Differences (mean ± 95%CI) between the peak period commencement time of univariate kinematic and mechanical performance variables and the perspective multifactorial metrics (shown as the reference point at time 0) for the match (a-b) and each half (c-d). Shaded areas represent the 5-min period *pre*- (occurring *before*) and *post*- (occurring *after*) peak period commencement time of the specific multifactorial variable (reference time 0 point). Effect size (*d*) 0.2–0.6, small (α).

Figure 2 presents the mean ±95%CI differences in the timing of occurrence between MDPk, MDPm and the MDP of their respective univariate constituent variables, across the match and within each half. Differences in the timing of occurrence considered the reference point (time 0) as the commencement time of either MDPk and MDPm, with positive and negative values indicating the occurrence of each univariate variable MDP after and before either MDPk or MDPm, respectively.

Overall, the timing of occurrence between MDPk and MDPm revealed small effect size differences in the overall match analysis (-375.67 ± 222.4 s (*d*:0.25 ± 0.15)). Trivial differences were observed in the analysis of the first half (-122.14 ± 109.1 s (*d*: 0.17 ± 0.15)), and second half (-101.0 ± 97.8 s (*d*:0.15 ± 0.14)). The results indicate that overall MDPm tend to occur after MDPk with the ± 95%CI range differences of 444.8 s across the match and ~218 s for the first half and ~196 s for the second half.

The MDPk constituent variables SPR and HSR occurred outside of the 5 min periods surrounding the timing of occurrence of MDPk, while the MSR occurred within the 5 min periods. Small significant differences were observed between MDPk and SPR (496.99 ± 224.2 s (*d*: 0.33 ± 0.2–0.5)), and HSR (379.01 ± 177.98 s (*d*: 0.26 ± 0.13–0.38)). No significant differences were observed between MDPk and MSR (Figure 2a). The MDPm constituent variables occurred within the 5 min periods surrounding the timing of occurrence of MDPm. Trivial differences were observed between MDPm and both ACC (117.19 ± 187.7 s (*d*: 0.08 ± -0.1–0.2) and DEC (-36.90 ± 66.1 s (*d*: 0.02 ± -0.1–0.13)) across the match (Figure 2b).

The analysis between halves revealed that the MDPk constituent variables occurred within the 5 min periods surrounding the timing of occurrence of MDPk, with differences in time smaller in the second half compared to the first half. Specifically, the 95%CI range of the time difference between MDPk and the respective kinematic constituent variables (MSR, HSR, and SPR) decreased from 383.3 s in the first half to 193.8 s in the second half, indicating closer proximity in their occurrences in the second half. Small significant differences were observed between MDPk and HSR (first half: 165.14 ± 83.6 s (*d* ± 95%CI: 0.21 ± 0.1–0.3); second half: 84.42 ± 78.4 s (*d*: 0.12 ± 0.01–0.2)) and SPR (first half: 161.22 ± 106.2 s (*d*: 0.21 ± 0.1–0.4); second half: 93.14 ± 91.2 (*d*: 0.14 ± 0.00–0.3)) (Figure 2c).

In line with previous results, the MDPm constituent variables occurred within the 5 min periods surrounding the timing of occurrence of MDPm, with differences in time smaller in the second half compared to the first half. The 95%CI range of the time difference between MDPm and the respective mechanical constituent variables (ACC and DCC) slightly increased between halves (first half: 226.9 s; second half: 292.3 s). However, no significant differences were observed between the time of MDPm and ACC (first half: 52.78 ± 89.4 s (*d*: 0.07 ± -0.1–0.20); second half: -67.96 ± 85.2 s (*d*: 0.09 ± -0.0–0.2)) and DEC (first half: -11.26 ± 73.49 s (*d*: 0.02 ± -0.1–0.12); second half: 75.38 ± 63.80 s (*d*: 0.11 ± 0.02–0.2)) within each half (Figure 2d).

### Magnitude

Differences in magnitude between MDPk, MDPm and the MDP of their respective univariate variables are presented in Table 1. Generally, the magnitude of the MDPk and MDPm in comparison with MDP univariate variables were small (p ≤ 0.001; *r*: 0.14–0.30) across the match. The percentage differences between multifactorial and discrete performance variables were approximately MSR: 15.0%; HSR: 19%; SPR: 39%; ACC: 29%; and DEC: 8%, with greater values observed for the univariate MDP metrics. MDPk was composed of approximately ~58% MSR, ~30% HSR, and ~12% SPR, while the percentage composition of MDPm was approximately ~36% acceleration and ~64% deceleration.

**TABLE 1:** Comparison (mean ± standard deviation (%CV)) between the MDP of multifactorial variables and their respective constituent variables for the match and each half.

Metric	Period	Match	First Half	Second Half
**MDPk (m)**		221.05 ± 53.62 (24.30%)	210.91 ± 56.37 (26.70%)	191.51 ± 51.55 (26.90%) **β

**MSR**	**MDPk (m)**	129.34 ± 40.27 (31.10%) ##α	126.52 ± 40.33 (31.90%)	112.20 ± 34.18 (30.50%) **β
	**MDP (m)**	148.74 ± 35.85 (24.10%)	141.63 ± 36.14 (25.50%)	126.51 ± 33.09 (26.20%) **β

**HSR**	**MDPk (m)**	69.64 ± 31.46 (45.20%) ##α	60.87 ± 27.32 (44.90%)	57.45 ± 27.26 (47.40%) **α
	**MDP (m)**	83.19 ± 27.98 (33.60%)	73.46 ± 26.00 (35.40%)	69.09 ± 24.89 (36.00%) **α

**SPR**	**MDPk (m)**	29.78 ± 18.81 (63.16%) ##α	28.76 ± 22.6 (78.56%)	28.34 ± 22.49 (79.35%)
	**MDP (m)**	41.30 ± 22.28 (53.94%)	35.57 ± 23.71 (66.68%)	32.64 ± 21.97 (67.31%)

**MDPm (m · s** ^−2^ **)**		0.066 ± 0.019 (29.50%)	0.061 ± 0.019 (31.70%)	0.055 ± 0.017 (31.30%) **α

**ACC**	**MDPm (m · s** ^−2^ **)**	0.024 ± 0.012 (50.00%) ##α	0.021 ± 0.011 (50.50%)	0.020 ± 0.010 (53.10%) *
	**MDP (m · s** ^−2^ **)**	0.031 ± 0.010 (32.80%)	0.026 ± 0.010 (37.50%)	0.025 ± 0.009 (37.70%) *

**DEC**	**MDPm (m · s** ^−2^ **)**	-0.044 ± 0.016 (35.62%) ##α	-0.039 ± 0.014 (36.80%)	-0.036 ± 0.013 (37.60%) **α
	**MDP (m · s** ^−2^ **)**	-0.047 ± 0.015 (31.31%)	-0.043 ± 0.013 (29.70%)	-0.039 ± 0.013 (33.30%) **α

## p ≤ 0.001; # p ≤ 0.05, significantly different than discrete univariate MDP.

** p ≤ 0.001;

* p ≤ 0.05, significantly different than the first half.

Effect size (*r*): 0.1–0.3, small (α) and 0.3–0.5, moderate (β). Data presented as mean ± standard deviation (%CV).

The magnitudes of MDPk and MDPm decreased significantly between halves (~9–10%), with small to moderately greater values observed in the first half (p ≤ 0.001; *r* = 0.24–0.30). There were no significant interaction effects for *half* × *period* across all performance variables. However, small to moderately significant (p ≤ 0.001; *r* = 0.26–0.44) main effects were observed for periods, indicating that the peak magnitude of the MDPk and MDPm were lower than their respective univariate metrics across halves. Differences between the MDPk and MDPm and the MDP of their respective univariate metrics were approximately: MSR: 12%; HSR: 21%; SPR: 20%; ACC: 26%, and DEC: 9%.

Significant (p ≤ 0.001) main effects were also observed for halves, with small significant decreases (p ≤ 0.001; *r* = 0.11–0.28) observed in the second half for all variables, except for SPR (p > 0.05) and ACC (p = 0.01, *r* = 0.07). Differences between halves were similar for MDPk and MDPm, by approximately 9%.

## DISCUSSION

In this study, a multifactorial analysis approach based on capturing concurrent MDP of discrete high-intensity kinematic (MDPk) and mechanical (MDPm) variables within match dynamics, is suggested. This study proposes and tests a multifactorial analysis to identify and characterize 5 min MDP of high-intensity activity in elite male football based on the concurrent occurrences of kinematic (MDPk) and mechanical (MDPm) performance variables. To the best of our knowledge, this study is the first to attempt the identification of MDPk and MDPm periods within multifactorial MDP performance variables. Our key findings demonstrate that the 5 min MDP are distinguished through peak kinematic and mechanical demands, which occur at distinct periods with unique locomotor profiles across both the match and each half. In line with our hypotheses the temporal distribution for MDPk and MDPm revealed greater frequency of MDP cases occurring at the start of the match and within each half. Regarding the timing of the MDP between multifactorial and respective univariate constituent variables, a closer proximity of occurrences was observed for mechanical than kinematic variables across the match. The magnitude of univariate MDP were greater than their respective constituent counterparts within the multifactorial MDP, however only by small effect size differences. Overall, our results highlight that the selection of multifactorial MDP criterion variables has implications in terms of its distribution throughout the match, as well as the timing and magnitude differences between its constituent variables and their univariate MDP counterparts.

The frequency analysis of multifactorial MDP aligns with previous research that explored the temporal distribution of MDP in elite football, although our approach considered kinematic and mechanical MDP as multifactorial performance variables, MDPk and MDPm respectively [4, 10, 12, 14, 15, 16]. Both MDPk and MDPm frequencies peak in the initial first 5 minutes of the match and the first half, however, the distribution patterns differ markedly in the second half. Notably, MDPk frequency peaks in the second 5 minutes of the second half. The MDPk distribution may be related to decreased muscle temperature during half-time intervals [28], impacting activities requiring a high rate of force development [29], such as sprinting. Accordingly, research by Novak et al. [16] associated 3 minute peak total distance and sprinting distance with earlier and later occurrences in the match and half, respectively, while peak high-speed running distance was more uniformly distributed. Fransson et al. [10] also observed similar distribution patterns for high velocity running distances across varying time periods. Collectively, these findings suggest that high velocity kinematic thresholds distribute more uniformly compared to lower-velocity thresholds [4, 10, 12, 14, 15, 16].

In contrast, MDPm demonstrates a more uniform distribution in the second half, potentially linked to the elevated metabolic cost and mechanical load associated with intense acceleration and deceleration efforts [19, 25, 30–32]. Players might strategically pace themselves and perform such efforts based on match context and at lower velocities (< 15 km/h), which might not coincide with the high velocity running efforts encompassed in MDPk. Correspondingly, a study of elite Australian soccer players demonstrated that ~85% of maximal accelerations did not surpass high-velocity thresholds (> 15 km/h) [25]. Therefore, variations in match running performance distribution could stem from the association of high-intensity actions with critical moments in the game, such as the creation or defense of goal-scoring opportunities [4]. Moreover, player-adapted pacing strategies may prioritize energy conservation for high-intensity runs at the expense of lower-velocity efforts [33]. As such, greater running distances at moderately high speeds early in the match may be strategic, while higher-velocity threshold efforts could be context-dependent throughout the game.

Comparisons of the timing occurrences between multifactorial (MDPk and MDPm) and their respective univariate constituent variables (MSR, HSR, SPR, ACC and DEC) revealed a decrease in the differences between kinematic variables between halves. Conversely, the time of occurrence between mechanical variables remained relatively consistent within each half and the match. Therefore, unlike kinematic variables, mechanical univariate peak periods aligned within the 5 min windows surrounding MDPm across the match and halves. However, the greater alignment of kinematic variables in the second half suggests that their clustering may be related to changes in match context, pacing strategies, or exercise tolerance, which may enable players to conserve energy for high-intensity activity [4, 33].

Comparative analysis between multifactorial and univariate performance variables indicated that the peak 5 min MDP magnitude was slightly greater when analyzed discretely, displaying small to moderate effect sizes, with reductions across the match and halves. Such difference could potentially be attributed to variations in MDP match dynamics between kinematic and mechanical variables. Discrete activities may be performed with greater intensity when examined individually rather than as multifactorial variables. For example, the higher magnitude of HSR may be due its occurrence at a greater intensity (or perhaps duration) elsewhere during the match to when the specified HSR (> 19.8–25.2 km/h) thresholds are concurrently considered with MSR (15–19.8 km/h) and SPR (≥ 25.2 km/h) thresholds within MDPk. This observation is further supported by the timing differences between MDPk and HSR, throughout the match. Such discrepancies might arise from methodological differences in identifying the MDP match running performance, especially concerning threshold selection, and moving average durations [7, 11, 10, 15]. These outcomes necessitate further investigation into the practical significance of magnitude differences between univariate and multifactorial performance variables.

Interestingly, the magnitude of all multifactorial and univariate constituent variables, except for SPR, decreased significantly between halves. The relative maintenance of SPR between halves may indicate that observed reductions in variables might not be solely due to physiologically mediated fatigue alone but may also be influenced by pacing strategies and match context [33]. The decrease in the magnitude of peak kinematic and mechanical performances between halves should be approached cautiously, as factors such as variations in effective playing time and periods of inactivity can influence these reductions [34, 35]. To attribute any performance reductions to fatigue in relation to observed changes, future studies should account for the specific relative time of play and players’ work rate [34].

The analysis also reveals high variability associated with the timing and magnitude of the presented variables. Examination of time differences between the commencement of multifactorial and univariate MDP variables demonstrates exceptionally high variability. For example, the variability between MDPk and the MDP of univariate kinematic constituent variables in the first half, across 45 matches, were 1660% (MSR), 757% (HSR), and 533% (SPR). In terms of magnitude, the variability for MDPk and MDPm across the match, and the halves, were similar. However, multifactorial constituents exhibit greater variability compared to their univariate counterparts across the match and halves, with SPR displaying the highest variability. Despite differences in the window durations investigated, our results are comparable with and exhibit similar trends to previously reported variability of peak 3 min univariate variables [16], such as HSR and SPR. These disparities likely result from distinct match-specific contextual variables and tactical roles that underlie players’ unique locomotor patterns. The high variability observed in time differences between the times of occurrence of the 5 min peak multifactorial and their respective univariate constituent variables underscores the non-concurrent nature of MDP across players, a notion previously highlighted [16].

The distinct approach of the study lies in considering the concurrent occurrence of univariate kinematic and mechanical performance variables as multifactorial variables, providing a robust means of identifying the 5 min peak locomotor performance during match play. However, the selection of multifactorial constituent variables may be related to the specific team or individual players analyzed, as they may differentially influence the distribution, magnitude, and composition of the respective multifactorial variables. For instance, while the peak multifactorial kinematic performance variable (MDPk) consisted of approximately ~58% MSR, ~30% HSR, and ~12% SPR running distances, and the peak multifactorial mechanical variable (MDPm) was composed of ~36% acceleration and ~64% deceleration, this may not remain constant across the match, for all players, and all observed MDP. Our findings, indicating changes between halves across all variables, reinforce the notion that the degree of constituent variable contribution to the respective MDPk and MDPm composition may fluctuate throughout the match, and may be influenced by other contextual factors. Differences and changes in terms of constituent variables could potentially influence their match distribution. Thus, future investigations should analyze the temporal distribution and percentage composition of peak multifactorial locomotor activities across the match to gain a more comprehensive understanding of dynamic locomotor performance patterns and how specific contextual variables can influence the distribution and magnitude of multifactorial constituent variables. Moreover, the practical significance of magnitude differences between univariate and multifactorial performance variables requires further exploration. The trade-off is that while our multifactorial analysis provides a robust approach to identifying the MDP through the concurrent occurrence of specific high-intensity kinematic and mechanical demands – which more accurately reflects competitive match scenarios – the discrete univariate analysis of MDP may be more relevant for precisely targeting specific locomotor activities.

Consequently, future studies and practitioners should consider adopting a multifactorial approach to identify kinematic and mechanical MDP across different durations and univariate constituent variables. The influence of univariate constituent variables on multifactorial MDP distribution profiles requires further investigation. It is plausible that the composition and degree of contribution of specific univariate constituent variables within multifactorial variables may vary temporally and be influenced by tactical and contextual variables during the match. For example, recent studies have highlighted that peak locomotor activities are significantly influenced by various contextual factors (e.g., tactical positions, match half, match outcome, tactical formation, possession, and phases of the game), which were not considered in the current study [13, 35–38]. Understanding the dynamics of multifactorial MDP composition profiles could further inform training prescription by enhancing ecological validity and representative design. Such adjustments may facilitate the implementation of specific task constraints in conditioning games to replicate match demands more accurately [9, 17, 20]. The incorporation internal load response (e.g., heart rate and sessional rate of perceived exertion (s-RPE) and individualized locomotor thresholds, which were not considered in our study, would further our understanding of the individualized performance and response to peak periods during competition [2, 16]. It is also imperative to acknowledge and account for sources of variability in future studies, incorporating a larger number of teams and players to enhance the generalizability of the findings. An additional limitation of our study was the analysis of two teams from the same professional league, and thus practitioners should take caution when interpreting the results. Lastly, the proposed approach underscores that kinematic and mechanical MDP in football are multifactorial and can be holistically analyzed to provide more effective insights for team and player performance monitoring and training prescription.

Therefore, the current study supports the use of multifactorial kinematic and mechanical MDP performance variables composed of relevant univariate performance variables to enhance the monitoring and prescription of training sessions. Such an approach allows the monitoring of several relevant independent (univariate) variables simultaneously. Practitioners may benefit from such an approach as the multifactorial MDP variables reflect the concurrent occurrence of demands across multiple velocity thresholds (kinematics) or considers both high-intensity accelerations and decelerations (mechanical). Prescribing training based on the MDP of match play through conditioning drills, such as small to large-sided games, may therefore be more effectively monitored as changes in the respective multifactorial MDP composition can be assessed to better target specific locomotor activities [9, 17, 18, 20, 35]. For example, a recent study by Martin-Garcia et al., [20] reported that specific locomotor activities are differentially trained (stimulated) depending on the format of conditioning drills based on match MDP, whereby small format games stimulate mechanical activity (acceleration and deceleration), while high-speed running and sprinting activities are stimulated in large format games. Practitioners may extend the findings in this study to better understand how the multifactorial MDP and its corresponding composition, regarding the constituent variables, change through consecutive drills. Monitoring the timing and changes in the magnitude and composition of kinematic and mechanical multifactorial MDP can provide further insight surrounding the development of transient fatigue during the match and residual fatigue and recovery strategies following the match.

## CONCLUSIONS

In conclusion, this study introduces a novel approach that considers the concurrent occurrence of univariate kinematic and mechanical performance variables as multifactorial variables, namely MDPk and MDPm. Our findings contribute to understanding that peak locomotor demands can be distinguished based on kinematic and mechanical profiles which are multidimensional, reflecting the concurrence of various locomotor activities. The influence of univariate constituent variables on multifactorial metrics’ frequency distribution as well as the composition may shift over time. Thus, the selection of univariate metrics could influence the contribution and composition of peak multifactorial kinematic and mechanical performance variables throughout the match. Identifying changes in the multifactorial metrics’ constituent variables may be relevant for targeting specific locomotor activities and identifying their influence on transient and residual fatigue development. Ultimately, this approach contributes to a more precise understanding of peak locomotor activity patterns, informing player performance monitoring and training design.
